# Tracking the shape-dependent sintering of platinum–rhodium model catalysts under operando conditions

**DOI:** 10.1038/ncomms10964

**Published:** 2016-03-09

**Authors:** Uta Hejral, Patrick Müller, Olivier Balmes, Diego Pontoni, Andreas Stierle

**Affiliations:** 1 Deutsches Elektronen-Synchrotron (DESY), NanoLab, Notkestrasse 85, D-22607 Hamburg, Germany; 2 Universität Hamburg, Fachbereich Physik, Jungiusstraße 9, 20355 Hamburg, Germany; 3 Universität Siegen, Fachbereich Physik, Walter-Flex-Straße 3, 57072 Siegen, Germany; 4 MAX IV Laboratory, Fotongatan 2, 22594 Lund, Sweden; 5 ESRF - The European Synchrotron, Radiation Facility, 71 Avenue des Martyrs, 38043 Grenoble, France

## Abstract

Nanoparticle sintering during catalytic reactions is a major cause for catalyst deactivation. Understanding its atomic-scale processes and finding strategies to reduce it is of paramount scientific and economic interest. Here, we report on the composition-dependent three-dimensional restructuring of epitaxial platinum–rhodium alloy nanoparticles on alumina during carbon monoxide oxidation at 550 K and near-atmospheric pressures employing *in situ* high-energy grazing incidence x-ray diffraction, online mass spectrometry and a combinatorial sample design. For platinum-rich particles our results disclose a dramatic reaction-induced height increase, accompanied by a corresponding reduction of the total particle surface coverage. We find this restructuring to be progressively reduced for particles with increasing rhodium composition. We explain our observations by a carbon monoxide oxidation promoted non-classical Ostwald ripening process during which smaller particles are destabilized by the heat of reaction. Its driving force lies in the initial particle shape which features for platinum-rich particles a kinetically stabilized, low aspect ratio.

## Abstract (web summary)

Understanding nanoparticle sintering is crucial for designing stable catalysts. Here, the authors use high energy grazing incidence X-ray diffraction as an *in situ* probe to track the compositiondependent three-dimensional restructuring of supported alloy nanoparticles during carbon monoxide oxidation.

## Graphical abstract

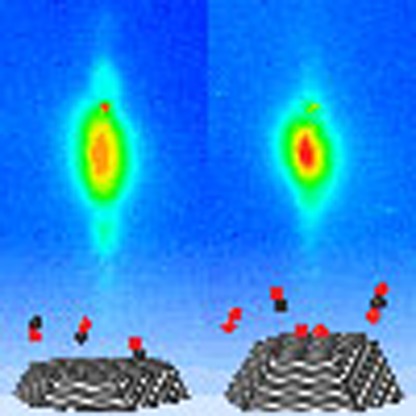

Heterogeneous, nanoparticle based catalysts are widely used in chemical industry as well as automotive exhaust converters or fuel cells for energy conversion. A major challenge are the harsh conditions during catalytic reactions since they often lead to nanoparticle sintering, which results in catalyst deactivation primarily due to the loss of active surface area^1^,^2^,^3^,^4^. Thus, research increasingly focuses on unravelling opportunities to avoid sintering mainly by particle encapsulation or size distribution tuning^5^,^6^,^7^,^8^,^9^. Alloy and bimetallic nanoparticles offer promising routes to mitigate particle sintering, in addition to tailored selectivity and activity due to ligand effects between neighbouring atoms^10^,^11^,^12^,^13^,^14^,^15^. The beneficial effect of alloying for sinter prevention under reaction conditions is however not well understood. Although much effort has been put into the study of sintering reversal via redispersion, its application is limited to certain systems and often requires the presence of undesired chlorine^16^,^17^, emphasizing the strong need to develop strategies for the production of more sinter resistant catalysts.

To reduce or avoid nanoparticle sintering an atomic scale understanding of the underlying mechanisms is mandatory. Two scenarios are proposed in the literature for thermally induced sintering processes^18^,^19^,^20^,^21^,^22^: (1) Ostwald ripening, in which larger nanoparticles grow at the expense of smaller ones by diffusion of single atoms or complexes driven by a gradient in chemical potential; (2) thermally activated complete particle migration and coalescence. Both processes get more complex when surrounding gas atmospheres are involved. Previous *ex situ* and *in situ* studies focused mainly on the sintering of nanoparticles induced by elevated temperatures and exposure to individual gases such as O_2_, H_2_, CO and H_2_O vapour or air^23^,^24^,^25^,^26^. In the case of Au nanoparticles on TiO_2_ a synergistic effect of CO and O_2_ for enhanced sintering under CO oxidation reaction conditions was reported^25^. Under pure CO atmospheres at 10^−4^ mbar platinum (Pt) nanoparticles on MgO(100) with diameters above 3.6 nm are observed to be stable up to 700 K (ref. 27). Recent density functional theory calculations demonstrated that CO can disintegrate 2 nm rhodium (Rh) nanoparticles via complex formation^28^.

Here we report a systematic study of the composition-dependent sintering, shape and size changes of epitaxial Pt–Rh alloy nanoparticles on Al_2_O_3_(0001) under catalytic CO oxidation reaction conditions close to atmospheric pressures. We record large area reciprocal space maps using high-energy grazing incidence x-ray diffraction as an *in situ* probe, and we use a combinatorial sample with composition gradient, assuring identical experimental conditions for different Pt–Rh compositions. Switching from reducing conditions to catalytic activity for CO oxidation is followed by *in situ* mass spectrometry. We find a composition-dependent nanoparticle sintering behaviour under near-atmospheric pressure CO oxidation conditions, which we attribute to a kinetically stabilized initial shape of more Pt-rich particles after their growth. Such a shape-dependent sintering mechanism has to our knowledge not yet been reported in literature.

## Result

### Measurement principle and sample design

**Figure 1:**
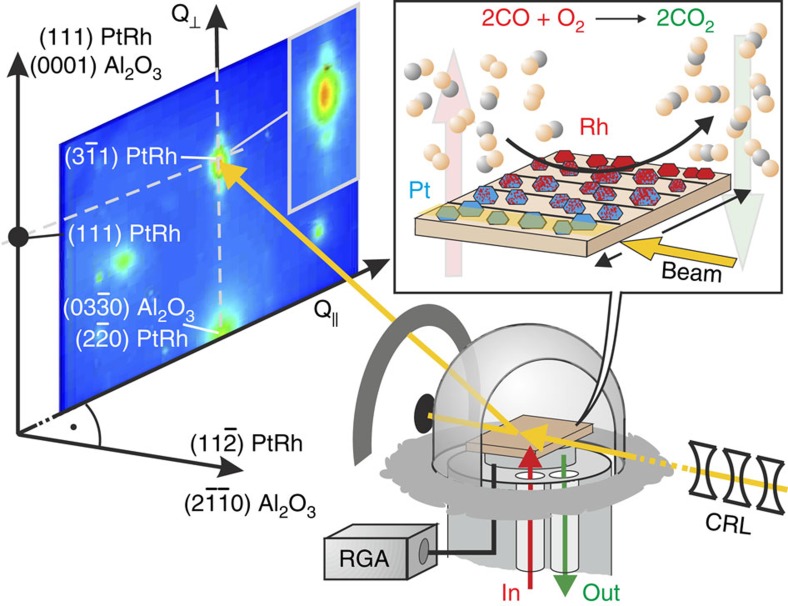
Measurement principle. The high-energy x-ray beam is focused by compound refractive lenses (CRLs) on the sample surface containing pure Pt, Pt_0.85_Rh_0.15_, Pt_0.7_Rh_0.3_, Pt_0.5_Rh_0.5_ and pure Rh nanoparticle stripes. The diffraction pattern is collected by a 2D detector while the heated sample is exposed to a computer-controlled gas flow mixture. The composition of the gas phase is controlled by leaking into a residual gas analyser (RGA). In the inset of the diffraction pattern a close-up of the 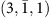 nanoparticle Bragg reflection is displayed.

The experiment was realized using a dedicated *in situ* x-ray diffraction chamber for catalysis experiments under flow conditions, as illustrated in Fig. 1 (ref. 29). The combinatorial sample contained stripes of Pt–Rh nanoparticles with varying composition from pure platinum to pure Rh and a constant height of ∼20 Å. The focused high-energy x-ray beam facilitated studying one stripe at a time and by translating the sample perpendicular to the incident beam nanoparticles of different Pt–Rh compositions could be probed subsequently under identical reaction conditions^30^,^31^. Monitoring the partial gas pressures inside the reactor allowed a direct correlation of particle structural changes to the catalytic activity^32^.

For all Pt–Rh compositions we find that the nanoparticles grow on the Al_2_O_3_(0001) substrate in (111) direction with distinct epitaxy as shown in Fig. 1. Note that the in-plane misfit between the alloy nanoparticles and the Al_2_O_3_(0001) substrate varies between +0.9% for pure Pt to −2.3% for pure Rh. Epitaxial nanoparticles, as employed in our study, give rise to well-defined x-ray diffraction Bragg peaks in reciprocal space with the absence of powder diffraction rings, see Fig. 1. While the particles' in- and out-of-plane lattice parameters are given by the Bragg peak position, the intensity distribution in the vicinity of the Bragg peaks holds quantitative information on the nanoparticle size and shape at the atomic scale^33^,^34^. To systematically unravel structural changes during CO oxidation we measured for each Pt–Rh composition and reactivity condition a two-dimensional (2D) reciprocal space map centred around the particle 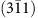 Bragg peak (Fig. 1). At high-photon energies the in-plane particle mosaicity of 1–2° probes a significant section of an azimuthal plane in reciprocal space and, therefore, gives rise to a diffraction pattern similar to transmission electron microscopy. The correlation between the wide-range detectability in reciprocal space and the particle mosaicity are illustrated and elucidated in Supplementary Fig. 1 and Supplementary Note 1. This allows a fast collection of large area reciprocal space maps without scanning sample or detector angles, which is crucial under reaction conditions^32^.

**Figure 2:**
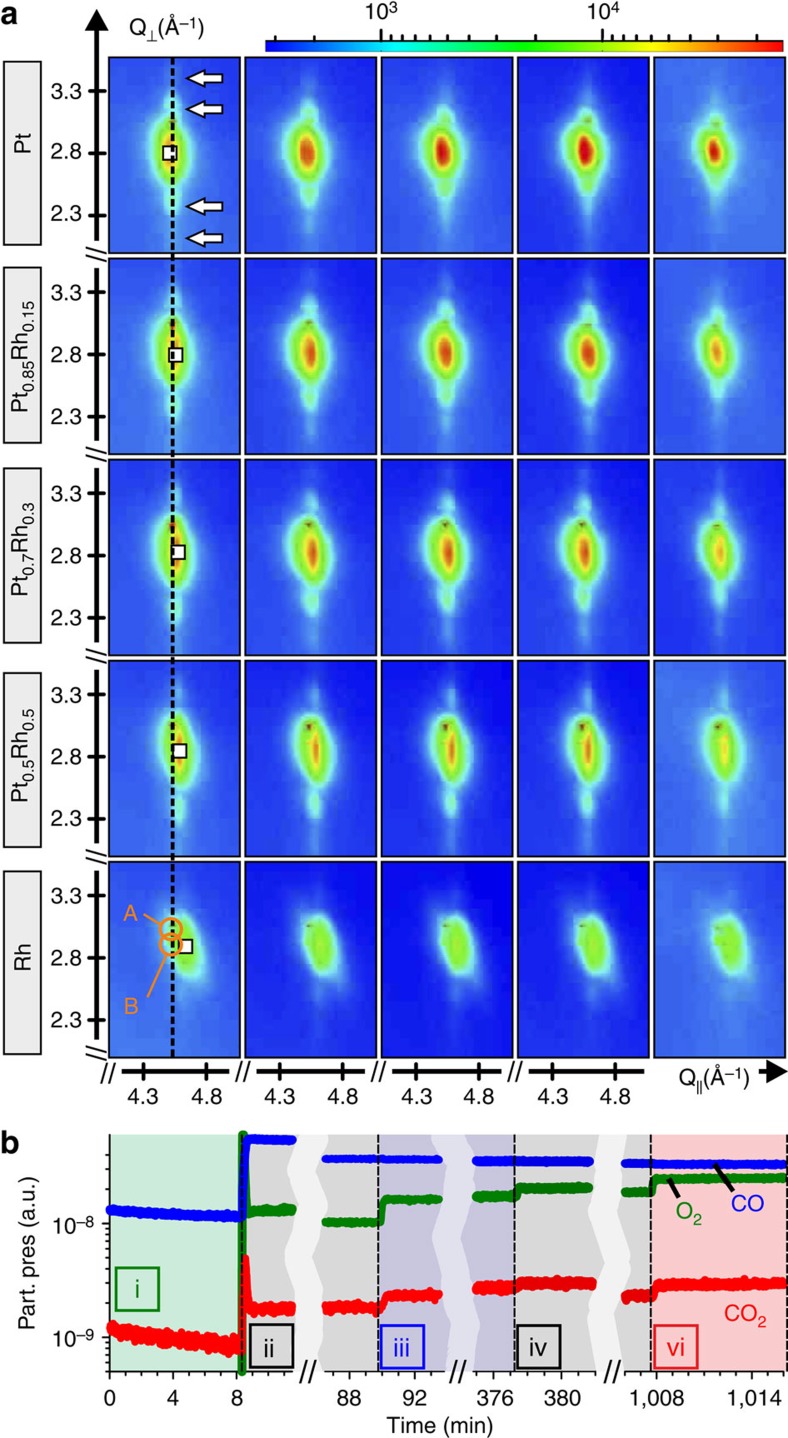
***In situ*** **monitoring of composition-dependent sintering during CO oxidation.** (**a**) Particle 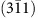 Bragg peak maps collected for different Pt–Rh compositions at 550 K and *p*_*tot*_=200 mbar when successively changing towards conditions of higher activity (i→vi; exposure time: 50 s). Set partial flows: i: flow *f*_*O*2_=0 ml min^−1^, *f*_*CO*_=2 ml min^−1^; ii: *f*_*O*2_=2 ml min^−1^, *f*_*CO*_=10 ml min^−1^; iii: *f*_*O*2_=4 ml min^−1^, *f*_*CO*_=10 ml min^−1^; iv: *f*_*O*2_=5 ml min^−1^, *f*_*CO*_=10 ml min^−1^; vi: *f*_*O*2_=7 ml min^−1^, *f*_*CO*_=10 ml min^−1^. Between iv and vi (not shown here): 2 cycles of gas switching between *f*_*O*2_=5 ml min^−1^, *f*_*CO*_=10 ml min^−1^ and *f*_*O*2_=0 ml min^−1^, *f*_*CO*_=2 ml min^−1^ (condition v, Fig. 3). Orange circles: position of substrate 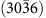 Bragg peak (A) and the intersection point of the substrate 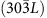 crystal truncation rod with the Ewald sphere (B). Vertical dashed line: in-plane substrate 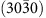 reference; white squares: positions of particle Bragg peak maxima. (**b**) Partial pressures of CO, O_2_ and CO_2_ as measured by the RGA under different conditons (i–vi) and as a function of time.

Figure 2a shows an overview of the 2D diffraction patterns for different Pt–Rh compositions (vertical panels) under the various conditions applied during the CO oxidation experiment (horizontal panels). The position of the Bragg peak maxima is given by Vegard's law according to their nominal composition, indicating that the as grown particles are present in the state of randomly mixed face centered cubic alloys. The intensity distribution around the particle Bragg reflections is smeared out in the *L*-direction perpendicular to the surface because of the finite particle height. It is characterized by periodical fringes above and below the Bragg peaks, especially pronounced for Pt-rich particles, indicated by arrows for the Pt particles in Fig. 2a. These Laue oscillations imply the presence of plate-like nanoparticles which are characterized by a low-defect internal structure and a small-vertical size distribution^35^.

### *In situ* monitoring of the composition-dependent sintering

In a first step the clean particles were probed under reducing atmosphere (condition i: *f*_*CO*_=2 ml min^−1^; no O_2_, *T*=550 K). For all subsequent settings (conditions ii–vi) the CO flow was kept constant at 10 ml min^−1^, as was the nominal temperature at 550 K, while the O_2_ flow was increased in steps up to 7 ml min^−1^. Ar was used as carrier gas adding up to a constant total flow of 100 ml min^−1^ at a constant total pressure of 200 mbar. The period of the Laue oscillations is very similar for all Pt–Rh compositions under condition i, implying that the particles of all stripes exhibited a very similar initial height. Figure 2b displays the residual gas analyser signal of CO, O_2_ and CO_2_ proportional to the respective average partial pressures inside the reactor for the aforementioned conditions. When switching to higher oxygen partial flows the CO_2_ production increased accordingly. Low-index Pt, Rh as well as Pt–Rh alloy surfaces and nanoparticles are reported to be catalytically active for CO oxidation at 550 K (refs 16, 36, 37), but since we measure the integral CO_2_ production over all stripes, we can not directly conclude which composition is the more active one. At 550 K and 20 mbar CO partial pressure (corresponding to our conditions) CO is expected to still partially cover the different Pt–Rh surfaces, in line with a Langmuir–Hinshelwood reaction mechanism^36^,^37^.

As key observation we note that the finite height fringes for Pt-rich particles progressively shift towards the Bragg peak position, when increasing the oxygen pressure and switching to higher catalytic activity (Fig. 2a). This implies that their height increases significantly when CO oxidation sets in because the *L*-spacing of their minima is inversely proportional to the particle height. We conclude that at same time the epitaxial arrangement of the particles is maintained since Debye–Scherrer powder diffraction rings are absent. It is striking that the shift of the fringes is progressively suppressed with increasing Rh compositions, which implies that sintering is strongly reduced for Rh-rich particles.

### Sinter-induced quantitative particle shape changes

**Figure 3:**
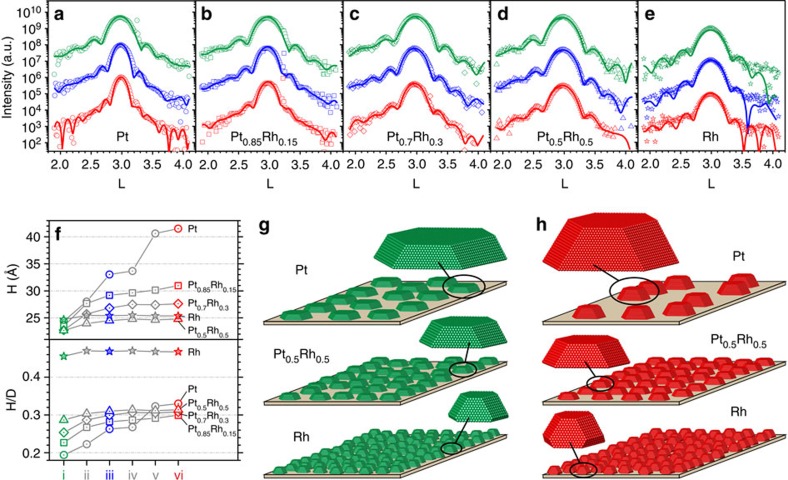
Sintering-induced particle shape changes for different Pt–Rh compositions. (**a**–**e**) Linescans in (111) direction (open symbols: data; solid lines: fit for underlying model particle shape with 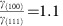) through the respective particle 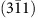 Bragg peaks extracted from the 2D maps of Fig. 2a for different Pt–Rh compositions ((**a**) Pt; (**b**) Pt_0.85_Rh_0.15_; (**c**) Pt_0.7_Rh_0.3_; (**d**) Pt_0.5_Rh_0.5_; (**e**) Rh) and various conditions (green: i; blue: iii; and red: vi). (**f**) composition-dependent particle heights and aspect ratios 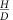 for different CO oxidation conditions (i–vi) as obtained from fits to the data, taking into account the average particle diameter as obtained by AFM after the experiment. The error bars are on the order of the symbol sizes. Average particle shapes and sample morphology before (**g**) and after (**h**) sintering as deduced from the Bragg peak and reflectivity fits are shown for some selected Pt–Rh compositions.

To obtain a deeper insight into the shape and size changes the particles underwent throughout the varying environmental conditions, we extracted the respective scattering profiles along *L* through the nanoparticle Bragg reflections from the 2D maps of Fig. 2a. The extracted rods were intensity corrected for the detector background, the missing *θ*-rotation of the stationary sample and the Lorentz factor, as is thoroughly described in Supplementary Note 2. It gets evident, that the Laue oscillations are damped in a characteristic way, which we attribute to a variation in occupancy of the different atomic layers directly related to the particle shape. The generic shape of a (111) oriented nanoparticle consists of (111) top and bottom facets as well as (111) and (100) side facets (see Fig. 3g,h, neglecting higher index facets for the moment). The equilibrium shape of the particles can be constructed for a given ratio 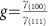 of the (100) and (111) surface energies *γ* (Gibbs free energy per surface unit, depending also on the surrounding gas atmosphere) by the Wulff-construction^38^. Geometrically meaningful values for *g* in the range from *g*=0.9 to 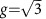
=1.73 were tested as starting models to fit the data, they are depicted in the top parts of Supplementary Figs 2 and 3 for the case of Pt_0.7_Rh_0.3_ particles under condition i. The truncation of the particles was performed in such a way that the observed finite height Laue oscillations were best reproduced, providing atomic layer sensitivity for the height determination. For a given particle shape, the different atomic layers exhibit a predetermined atomic occupancy, which leads to a characteristic damping of the Laue oscillations. In total, eight different starting models were tested for each condition and composition which were further refined during the fit in the following way: the occupancy numbers of the atomic layers at the substrate-particle interface and up to five layers on the top part of the particle accounting for particle height fluctuations were allowed to vary. Further fit parameters comprised the displacement parameters of these atomic layers. The fit parameters are depicted in Supplementary Fig. 4, the fitting procedure is elucidated in the Methods part at the end of this article.

As a general trend for all conditions and compositions, we obtain good fit results when starting with shapes of truncated (111)-oriented particles with ratios of 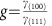 ranging approximately from 0.9 to 1.3, compatible with particles possessing distinct (100)-type facets (Supplementary Fig. 2). Particles with highly reduced (100)-type facets and nearly triangular shaped particles are unlikely to occur (*g*=1.5). Figure 3a–e shows the fits for *g*=1.1 to the experimental data for the different Pt–Rh compositions and conditions i, iii and vi. The three-dimensional (3D) models for particles for a starting model with *g*=1.1 are represented in Fig. 3g,h for some selected Pt–Rh compositions. Supplementary Note 3 contains a more thorough explanation of the fit results.

Under vacuum conditions, theoretical *g* ratios vary from 1.05 for Pt to 1.16 for Rh (refs 39, 40). In the case of CO and CO/O mixtures, no systematic investigation of the surface energy on Pt, Rh as well as Pt–Rh alloy surfaces as a function of coverage and chemical potentials is reported, which would allow a direct prediction of the particle shape using the Wulff construction. The fitted occupancies for the topmost particle layers (Supplementary Fig. 3 and Supplementary Note 3) show a reduction compared with the values for ideal particle shapes. This can be explained by a moderate particle height distribution and/or the presence of higher indexed facets, in-line with a slightly rounder particle shape^39^. Experimentally, the stabilization of low-index facets was observed during CO oxidation at 470 K by transmission electron microscopy^41^, supporting a moderate particle height distribution in our case.

From the fits we find that the occupancy parameters of the interface layer for nearly all compositions and conditions range between 0.4 and 0.7 (Supplementary Table 1 and Supplementary Note 3), respectively. This points to misfit induced defects at the interface to the substrate, which locally disturb the lattice. The interfacial occupancy values decrease after reaction-promoted sintering, pointing to a growing number of interfacial defects such as misfit dislocations in line with previous findings^42^. The first and second metal layer distance at the interface is found to increase by 0.1–0.2 Å, likely because of the interaction with the Al_2_O_3_ substrate (Supplementary Table 1).

**Figure 4:**
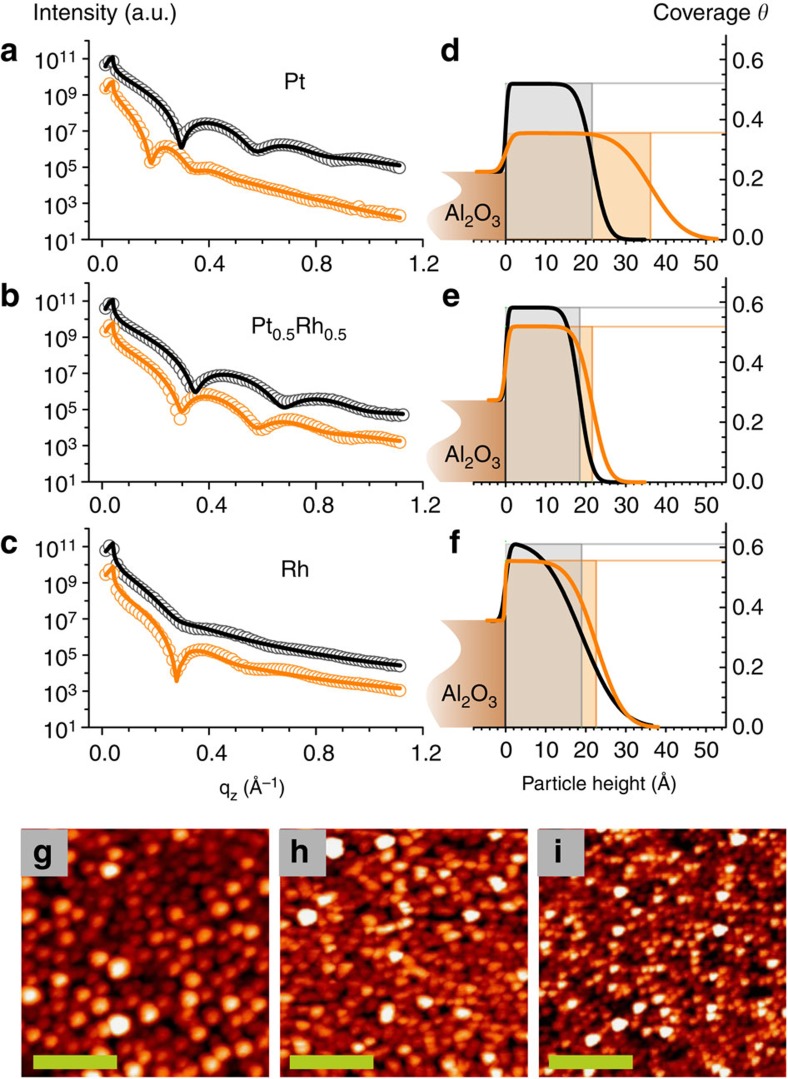
Composition-dependent particle height and coverage and diameter before and during CO oxidation. (**a**–**c**) x-ray reflectivity measurements (open circles: data, solid lines: fit) performed on particles with different Pt–Rh compositions and (**d**–**f**) electron density profiles obtained from the fit results which yield information on the particle height and percental particle coverage (black, before CO oxidation; orange, during CO oxidation at flows of 7 ml min^−1^ O_2_ and 10 ml min^−1^ CO). The shaded boxes represent the average particle height and coverage. (**g**–**i**) AFM measurements performed under UHV after the CO oxidation experiments: (**g**) Pt; (**h**) Pt_0.5_Rh_0.5_; (**i**) Rh. The scale bars correspond to a length of 100 nm.

Figure 3f summarizes the average particle height in the course of the CO oxidation experiment as obtained from the fit. While for pure Pt particles the height almost doubles changing from 24 to 42 Å; the height increase is reduced for the Pt_0.85_Rh_0.15_ particles changing from 23 to 31 Å, whereas the height of pure Rh particles stays nearly constant (26 Å compared with 24 Å). This composition-dependent discrepancy in height of the sintered particles is also confirmed by our atomic force microscopy (AFM) and x-ray reflectivity data (Supplementary Table 2, Supplementary Figs 5c and 6). At the same time, we estimate from rocking curve measurements that the average particle diameter *D* stays approximately constant within the error bars (*D*=12 nm for pure Pt, decreasing to 6 nm for pure Rh, our approach for deducing the particle diameter from rocking scans is illustrated and explained in Supplementary Figs 7,8 and Supplementary Note 4, respectively). The values at the end of the experiment are confirmed by AFM performed after the *in situ* experiments (Fig. 4g–i, Supplementary Fig. 5 and Supplementary Note 5), which in addition disclose a large size distribution (Supplementary Fig. 5b). For a second composition gradient sample which was investigated in the 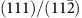 azimuthal plane (Supplementary Fig. 9) a similar behaviour was observed (Supplementary Fig. 10 and Supplementary Note 6). The results allow us to plot the nanoparticle average aspect ratio as a function of the reaction conditions (Fig. 3f), exemplifying a strong increase in aspect ratio for Pt-rich particles.

### Adhesion energy determination

The height of the particles depends under equilibrium conditions on the interfacial energy according to the Wulff–Kashiew theorem^43^. The determined particle shape, therefore, also holds information on the adhesion energy *W*_*adh*_, which can be calculated for strongly truncated nanoparticles based on the truncation of the particles and reference values for (111) and (100) facet surface energies^39^,^44^. A sketch of such a strongly truncated particle resembling the ones on our sample after sintering is depicted in Supplementary Fig. 11. Assuming the sintered particles at the end of the experiment have adopted equilibrium shape, we find the following adhesion energies taking a surface energy ratio of 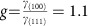 and the theoretical values for the clean surface energies *γ*_(111)_ into account: 2 J m^−2^ for pure Pt, 2.2 J m^−2^ for Pt_0.85_Rh_0.15_, 2.24 J m^−2^ for Pt_0.7_Rh_0.3_, increasing to a maximum of 2.6 J m^−2^ for Pt_0.5_Rh_0.5_ and decreasing again to 1.9 J m^−2^ for pure Rh (the systematic error bar of ±0.2 J m^−2^ takes the experimental uncertainty of *g* from 0.9 to 1.3 into account, see Supplementary Table 3 and the Methods section part at the end of this article for further explanations). The values presented here represent an upper limit, since *γ*_(111)_ may be slightly reduced in the presence of adsorbates under reaction conditions^39^. The adhesion energy values are compatible to literature values for Pd nanoparticles on ultrathin Al_2_O_3_ films^44^. The observed trend reflects the competing influence of the increasing chemical interaction from Pt to Rh for stoichiometric Al_2_O_3_ surfaces^45^, and the variation in misfit to the Al_2_O_3_ substrate. A perfect match to the substrate is expected for a composition of Pt_0.7_Rh_0.3_. For an increasing misfit to the Rh-rich side the adhesion energy is expected to decrease^42^,^46^. At the same time the chemical interaction is increasing, resulting in a shift of the maximum of the adhesion energy to a Rh composition of 50%. Such a competition between chemical interaction and misfit-induced deformation energy is expected also for other oxide supported metal nanoparticles, and allows to tune the adhesion of the nanoparticles to the oxide support.

### Mass transport on the sample surface during reaction

To further corroborate the observed sintering behaviour, *in situ* x-ray reflectivity experiments were performed before CO oxidation and under reaction conditions at flows of 7 ml min^−1^ O_2_ and 10 ml min^−1^ CO. X-ray reflectivity provides information on the average electron density profile perpendicular to the surface, related to the average particle height and the surface coverage on an absolute scale^47^. The x-ray reflectivity measurements performed before CO oxidation (black curves in Fig. 4a) confirm the initially similar particle heights of 18–22 Å and result in relative particle coverages on the different stripes of 50–60%, respectively. The qualitative inspection of the reflectivity curves in Fig. 4 (and also Supplementary Fig. 6) reveals that the period of the particle height-dependent interference fringes decreases much more strongly for pure Pt and Pt-rich particles, as compared with pure Rh. The fit reveals that the average height of the pure Pt particles increases from 22 to 36 Å, whereas the height increase is less pronounced for Pt_0.85_Rh_0.15_ particles (from 19–29 Å) and almost not present for particles with higher Rh composition (Pt_0.5_Rh_0.5_: from 18 to 22 Å, pure Rh: from 19 to 23 Å, Supplementary Table 2). The particle heights obtained from the reflectivity data are in general smaller than the ones deduced from the Bragg peak fits. This is due to the fact that the simple box model used for fitting the reflectivity data systematically underestimates the particle height, because it does not take into account the details of the particle shape^48^.

Interestingly, the height increase is found to be proportional to the reduction of the particle surface coverage, keeping the integral of the electron density profile constant within the error bars. The reduction in coverage is most pronounced for pure Pt particles for which it decreases from 52 to 35%, as can be inferred from the electron density profiles (Fig. 4d–f and Supplementary Fig. 6). The reflectivity results strongly suggest that a huge mass transport takes place on the sample surface during the activity-induced restructuring of the Pt-rich nanoparticles. In addition, they prove the conservation of catalyst material which is represented by the area of the shaded boxes in Fig. 4d–f (Supplementary Fig. 6) and which remains constant within error bars for all Pt–Rh compositions. Despite the tremendous material transport the sample features separated and well-defined nanoparticles, as can be inferred from the AFM images measured after the CO oxidation experiment (Fig. 4g–i and Supplementary Fig. 5a). The height histograms obtained from the AFM images (Supplementary Fig. 5b) correspond well to the roughness values determined by x-ray reflectivity, see Supplementary Table 2.

## Discussion

We now turn to the key question concerning the driving force for the observed 3D sintering behaviour of Pt-rich particles. According to our observations, Rh and Rh-rich particles essentially maintain their original size and shape under reaction conditions, which they had initially obtained after deposition at 800 K. From this we conclude that they are close to equilibrium shape. For Pt-rich particles the total surface coverage decreases under CO oxidation conditions, which implies a tremendous lateral inter-particle mass transport, as well as intra-particle mass transport. The Pt-rich particles undergo a significant change in aspect ratio, while keeping their average diameter approximately constant. As a realistic scenario, we propose that within the inherently large lateral size distribution (see AFM images in Fig. 4g–i and Supplementary Fig. 5a,b) smaller particles are present which are less stable. The energy released during the CO oxidation reaction on Pt is 3 eV per produced CO_2_ molecule^49^, which when locally thermalized may easily promote the destabilization of smaller particles. The observed phenomenon can thus be viewed as a CO oxidation promoted non-classical Ostwald ripening process during which as a net result larger Pt-rich particles essentially grow vertically. The net growth of larger Pt particles takes place predominantly perpendicular to the surface, as a result of the balance between the tendency of the particles to obtain a more 3D equilibrium shape and the lateral growth during the non-classical Ostwald ripening process. The particles overcome the kinetically stabilized particle shape with low aspect ratio towards a shape closer to thermal equilibrium. The CO oxidation thus locally releases energy opening new pathways for thermal atomic diffusion rendering this tremendous catalyst restructuring possible.

In summary, we monitored, operando, the composition-dependent sintering of alloy nanoparticles during catalytic CO oxidation, which we found to be more pronounced for Pt-rich particles. Rh-rich particles resist sintering under identical reaction conditions. We employed high-energy x-ray diffraction as *in situ* tool together with a combinatorial sample geometry, beneficial for efficient data acquisition. The restructuring is characterized by a strong particle height increase accompanied by a decrease of the particle surface coverage. For Pt nanoparticles, the addition of Rh gives rise to an advantageous higher dispersion and the increased mismatch to the substrate promotes a more 3D shape, closer to equilibrium. Our findings have implications for the preparation of more sinter resistant particles: to avoid shape transformations, particles with equilibrium shape are preferable, which are sufficiently large to sustain the reaction-induced local heat dissipation. A reduction in size distribution will be beneficial to reduce sintering, since it is expected to minimize the reaction-induced destabilisation of small particles. The reaction heat itself sets, however, a lower limit to the useful particle size.

## Methods

### Sample preparation

The Pt–Rh alloy nanoparticles were prepared by means of physical vapour deposition under ultrahigh vacuum (UHV) conditions (base pressure below 3 × 10^−11^ mbar) at a substrate temperature of 800 K. For the simultaneous evaporation of the two materials an Omicron EFM 3T evaporator was used. The particle stripes of varying composition were prepared successively by using a Ta mask with a slit in front of the sample. The calibrated Pt and Rh fluxes were in case of each stripe tuned according to the desired Pt–Rh composition. The stripes were grown perpendicular to the reciprocal space plane of interest (sample I: stripes along the 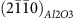
–direction to monitor the particle 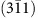 peak in the 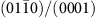 Al_2_O_3_–plane; sample II: stripes along the 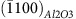
–direction to measure the particle 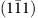 peak in the 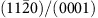 Al_2_O_3_–plane). Before deposition the Al_2_O_3_(0001) substrates had been chemically treated in an ultrasonic bath (15 min in acetone and 15 min in ethanol). Thereafter the substrates underwent cracking with atomic oxygen (*T*_*substrate*_=570 K; p_*O*2_=1 × 10^−7^ mbar; and *P*_*cracker*_=65 W) to remove residual carbon. The correct substrate orientation with respect to the slit position and the cleanliness of the surface before deposition were confirmed by low-energy electron diffraction and Auger electron spectroscopy, respectively.

### Experimental set-up and sample environment

The x-ray measurements were performed in a dedicated *in situ* catalysis chamber^29^ (base pressure: 2 × 10^−9^ mbar). Inside the chamber we annealed the samples before the CO oxidation experiments under hydrogen (*p*_*H*2_=2 × 10^−5^ mbar, *T*=490–540 K) to remove possible oxides since the samples had been exposed to air after growth. Apart from the use of CO and O_2_ we employed Ar as carrier gas to keep the total pressure and total flow inside the reactor at 200 mbar and at 100 ml min^−1^, respectively. The cleanliness of the CO gas was ensured by the use of a carbonyl trap inserted in the CO line before the reactor.

The x-ray measurements were carried out at the high-energy beamline ID15A (ESRF) at a photon energy of 78.7 keV. The 2D reciprocal space maps were measured with a FReLoN 14 bit CCD camera, x-ray reflectivity was performed using a NaI scintillation point detector. For imaging the 2D maps we used a shallow incident angle (*α*_*in*_=0.0333°) below the substrate critical angle to suppress scattering from the bulk. The beam was focussed by 200 compound refractive lenses which resulted in a vertical and horizontal beam size of 8 and 25 μm at the sample position. At *α*_*in*_=0.0333° this results in a beam footprint of 13.8 mm which covers the whole length of the particle stripes (sample dimensions: 10 × 10 × 1 mm^3^).

### Fitting the particle rods extracted from the 2D maps

The particle diffraction rods were extracted from the 2D maps parallel to the (111) direction. The obtained intensity distribution was corrected for the detector background, the missing *θ*-rotation of the stationary sample, and the Lorentz factor (Supplementary Note 2). They were simulated using the programme package of ROD (refs 50, 51), which allows to refine surface structure models, where the fitting parameters include among others occupancy parameters describing the percentaged atomic density of the different layers, as well as displacement parameters accounting for the relaxation of the atomic layers in vertical direction. In our approach we used the underlying surface structure model to mimick the nanoparticle structure: as the generic nanoparticle shape is determined by the atomic layer density profile in the vertical direction it was in our particle model shapes accounted for by adjusting the occupancy parameters for each atomic layer accordingly. Based on the Wulff-construction, different underlying particle shape models were put to the test in the particle rod fitting procedure (see next paragraphs as well as Supplementary Note 3, and Supplementary Figs. 2–4).

### Particle model shapes

The different underlying particle shape models used in the fitting procedure differed in the particle shape itself, but adhered for a certain condition and composition to the particle height *H* and diameter *D* deduced from the x-ray data on the basis of the finite thickness oscillations and/or the AFM measurements. The model shapes comprised eight truncated (111)-oriented particle models of varying surface energy ratios 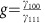 based on the Wulff-construction^38^, a truncated sphere model and a simple model of a continuous layer. A juxtaposition of the different model particle shapes is presented at the top of Supplementary Figs. 2 and 3 for the case of Pt_0.7_Rh_0.3_ particles under condition i (no O_2_, 2 ml min^−1^ CO, *p*_*tot*_=200 mbar and *T*=550 K).

### Fitting procedure

To illustrate the fitting procedure and the parameters varied therein Supplementary Fig. 4 shows as an example the fit results obtained for Pt_0.7_Rh_0.3_ particles under condition i. The particle height and diameter in this case comprised *H*=18 and *D*=90 *Å*, respectively, and the particle was accordingly concluded to consist of nine atomic layers. Supplementary Figure 4a shows the corresponding particle model for a surface energy ratio of *g*=1.1, Supplementary Fig. 4b the respective layer model. Each layer has an occupancy value determined from the corresponding atomic density, where the ninth and thus the topmost layer (occupancy value *t*_0_) is indicated by black dashes. The occupancy values according to the (unfitted) model shape of Supplementary Fig. 4a are represented by the red line in Supplementary Fig. 4c.

To compensate for the particle height distribution, up to two layers were added to the layer model. During the fit the occupancy values of these two layers (*t*_1_, *t*_2_), the ones of layers *t*_−1_, *t*_−2_, *t*_0_ and of the two layers at the interface (*b*_0_, *b*_1_) were allowed to vary. If the fit suggested unphysical values the number of parameters was reduced accordingly (for example, only one layer was added on top and only the occupancy values *t*_1_, *t*_0_, *t*_−1_, *b*_0_ and *b*_1_ were used as fit parameters, or even no layer was added on top and only the occupancy values *t*_0_, *b*_0_ and *b*_1_ were used as fit parameters). The values of the occupancy parameters were considered as physically meaningful as long as with increasing distance of the atomic layer from the particle bulk the corresponding occupancy values decreased (that is, *t*_−2_≥*t*_−1_≥*t*_0_≥*t*_1_≥*t*_2_ and *b*_1_≥*b*_0_).

Apart from the occupancy parameters also displacement parameters, which consider the layer relaxation, were included in the fit. Supplementary Figure 4c shows the fitted occupancy values for the case of the Pt_0.7_Rh_0.3_ particles under condition i (black diamonds). The fitted displacement parameters can be extracted from Supplementry Table 1.

### Adhesion energy determination

Assuming that after sintering (condition vi) the particles have adopted their equilibrium shape, the adhesion energy *W*_*adh*_ was deduced for the different Pt–Rh compositions.

Supplementary Figure 11 shows the side view of a strongly truncated particle, similar to the ones present on our sample, where the cross-section of the untruncated particle was obtained according to the Wulff-construction^38^. The height of the truncated particle is less than half the height of the unsupported particle and the effective surface energy γ* can be written as γ*=*γ*_*interface*_−*γ*_*substrate*_ (refs 43, 44). Knowing the particle height *H*, the length *w* of the top facet, the surface energy ratio 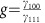 and the surface energy of the (111)-type facet this effective surface energy can be expressed as^44^:


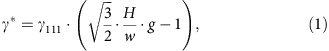


from which the adhesion energy *W*_*adh*_ can be determined:


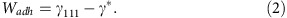


This formula was used to estimate the adhesion energy of the Pt-containing particles after sintering. In the case of the pure Rh particles formulas (1) and (2) could not be applied since the height of the truncated particles was higher than half of the height of the unsupported particles. Instead, the Wulff–Kaishew theorem, which can be expressed as


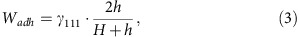


was employed, where *h* is the height of the ‘buried' part of the particle, as can be seen in Supplementary Fig. 11. The results for the adhesion energies *W*_*adh*_ for the particle shapes of the different compositions with a surface energy ratio of 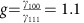 are written in bold and are summarized in Supplementary Table 3. The results for the values of *g*=0.9 and *g*=1.3 are included and can be considered as the maximum and minimum values of the respective error bars.

### Employed coordinate systems

For the metal nanoparticles face-centred cubic bulk reciprocal lattice indices are used (cubic room temperature lattice constants *a*_*Pt*_=3.924 Å and *a*_*Rh*_=3.801 Å) and for the Al_2_O_3_ substrate hexagonal reciprocal lattice indices (room temperature lattice constants *a*=*b*=4.763 Å, *c*=13.003 Å, *α*=*β*=90° and *γ*=120°).

### Fitting of the x-ray reflectivity data

The x-ray reflectivity data were corrected for the absorbers and the dead time of the point detector. In the fit model the layer of nanoparticles is represented by a closed box whose height is obtained from the fit. The interface roughnesses are in the underlying modified Parratt formalism taken into account as Gaussian fluctuations around the interface layers. Comparison of the fit parameter for the electron density with the corresponding density literature value for a closed layer yields the fraction of surface covered by nanoparticles. The fitting programme accounts for the angle-dependent beam footprint on the sample.

### AFM measurements

The AFM measurements were performed after the CO oxidation experiments under UHV conditions in non-contact mode using an Omicron VT AFM XA instrument.

## Additional information

**How to cite this article:** Hejral, U. *et al.* Tracking the shape-dependent sintering of platinum–rhodium model catalysts under operando conditions. *Nat. Commun.* 7:10964 doi: 10.1038/ncomms10964 (2016).

## Supplementary Material

Supplementary Information Supplementary Figures 1-11, Supplementary Tables 1-3, Supplementary Notes 1-6 and Supplementary References
