# Supplementary material for: Tracking the shape-dependent sintering of platinum–rhodium model catalysts under operando conditions
Source: Nat Commun. 2016 Mar 9;7:10964. doi: 10.1038/ncomms10964 (PMC4786879; doi:10.1038/ncomms10964)
Supplement: Supplementary Information — Supplementary Figures 1-11, Supplementary Tables 1-3, Supplementary Notes 1-6 and Supplementary References [file ncomms10964-s1.pdf]

Supplementary Figures

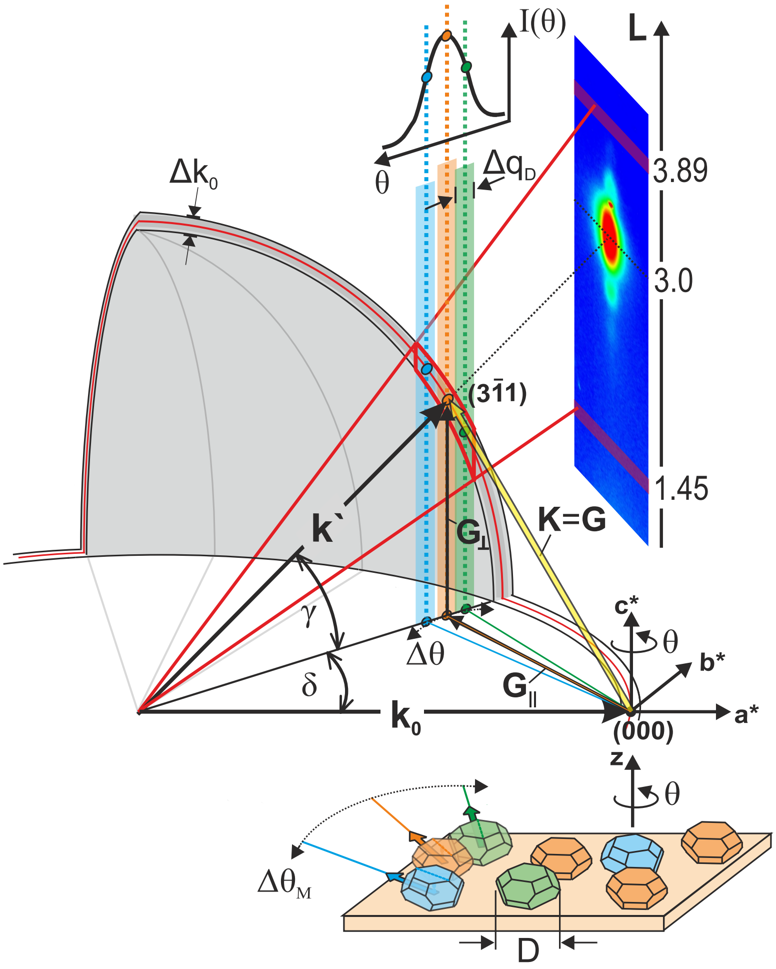

Supplementary Figure 1: **Measurement principle.** the angular distribution $\Delta\theta$ of the particles, the particles' average diameter D and the non-zero energy resolution $\Delta E_0 = \frac{\Delta \mathbf{k_0}}{\mathbf{k_0}} \cdot E_0$ lead to the detectability of a wide range of particle diffraction signal along the particle rod, which can be probed with a 2D detector snapshot at a fixed rocking angle θ.

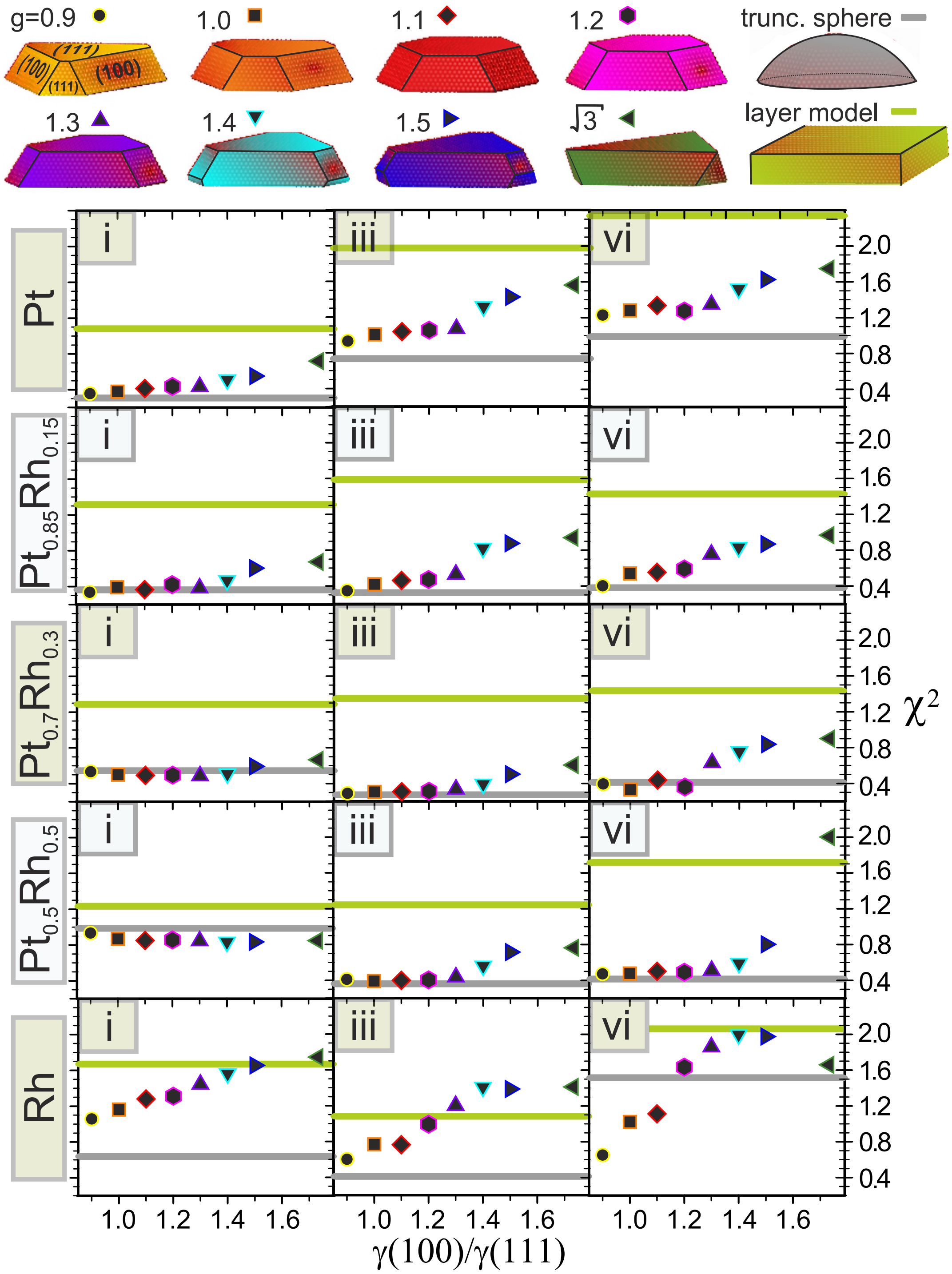

Supplementary Figure 2: **Goodness of fit for different model shapes:** comparison of figures of merit χ^2 obtained from fitting the particle rods using different underlying model shapes. For each Pt-Rh composition and condition (i, iii, vi) they are displayed for various particle facet surface energy ratios $g = \frac{\gamma_{(100)}}{\gamma_{(111)}}$ of the Wulff shape (represented by circles, squares, diamonds, hexagons and triangles; for colour coding see figure top). Horizontal grey and light-green lines represent figures of merit obtained for the case of a truncated sphere and a closed layer, respectively.

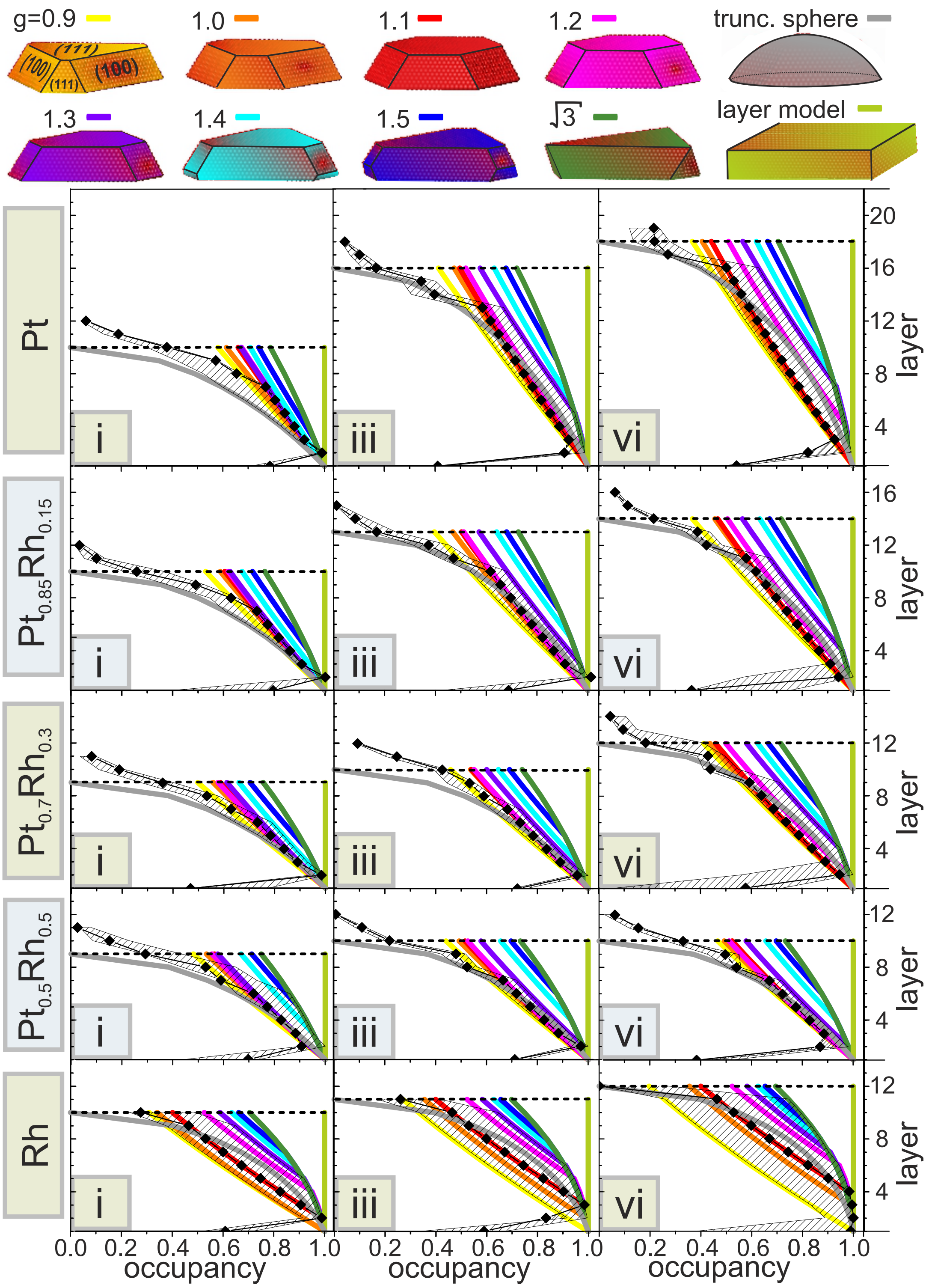

Supplementary Figure 3: **Comparison of occupancy values:** comparison of fitted occupancy values (black diamonds, based on the particle shape model with g=1.1) to theoretical occupancy values of other underlying model shapes (see figure top, also for colour coding). The black-dashed area illustrates the parameter space of fit results obtained from the five best fits of all tested g-values and of the truncated sphere model. The horizontal and black dashed line corresponds in each case to the particle height deduced from the x-ray data, the fitted occupancy values in comparison mirror the smearing out of electron density in vertical direction due to particle height distribution.

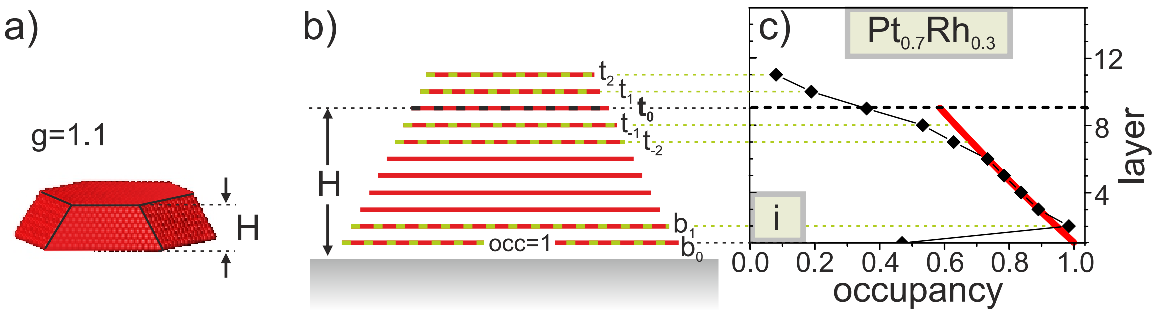

Supplementary Figure 4: **Particle models and fit parameters: (a)**: shape of the $Pt_{0.7}Rh_{0.3}$ particles under condition i with height H=18 $\mathring{A}$, diameter D=90 $\mathring{A}$ and surface energy ratio $g=\frac{\gamma_{001}}{\gamma_{111}}$=1.1. **(b)**: sketch of the corresponding fit model that clarifies the different fit parameters; the total number of layers is determined by the particle height H, as obtained from the finite thickness oscillations around the particle Bragg peak, where up to two additional layers (t_1, t_2) were added to compensate for the vertical particle size distribution. For the fit the occupancy values of layers t_2, t_1, t_0, t_{-1}, t_{-2}, b_1 and b_0 were allowed to vary as well as the displacement parameters of up to three topmost layers and up to two layers at the interface. **(c)**: occupancy values according to the underlying particle shape model displayed in **a** (red line) and fitted occupancy values (black diamonds). The text occ=1 in panel **b** indicates that in this example the theoretical occupancy for the lowest layer b_0 of the unfitted model is 100 %, where the occupancy values of the other layers are in this initial model normalized to this nominal occupancy. These unfitted values represent the ideal case depicted in **a**.

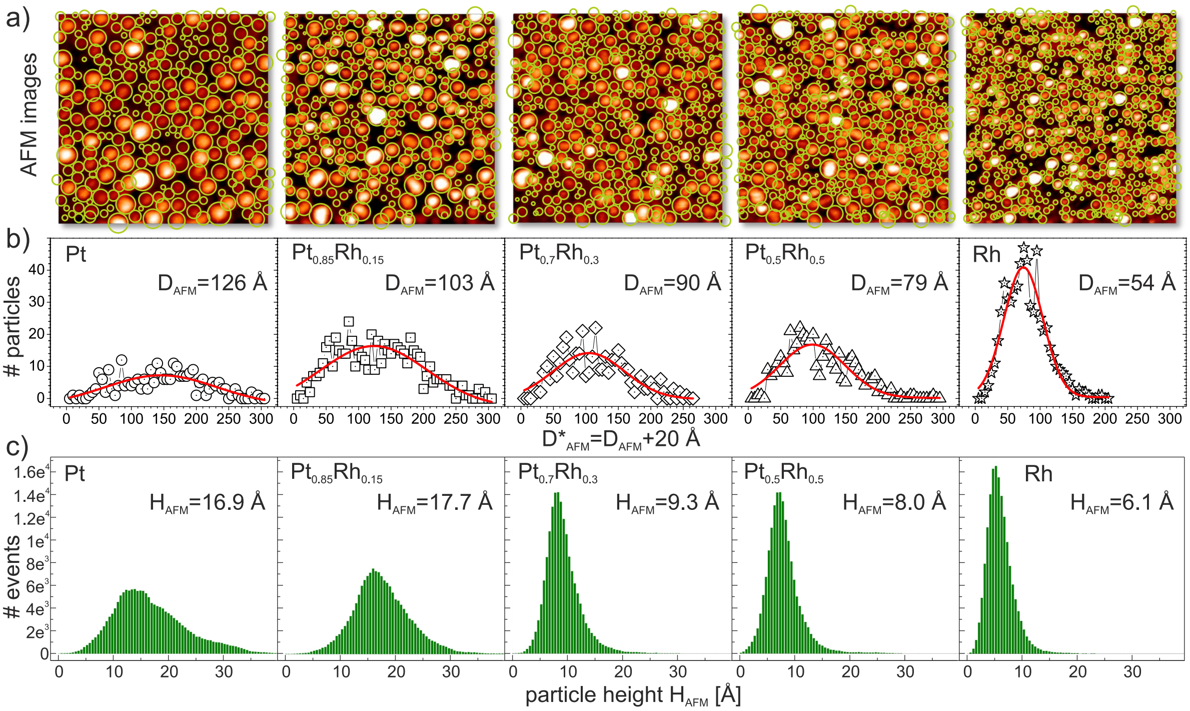

Supplementary Figure 5: **AFM measurements: (a)**: AFM images from particles with different Pt-Rh compositions. The green circles indicate the particle peripheries that were used for particle size distribution determination. **(b)**: deduced particle horizontal size distribution, the peak maxima represent the average particle diameter D_{AFM} that was used for the particle rod analysis. **(c)**: height histogrammes as obtained from the AFM data shown in the top panel. The values of the average heights are included. They are also summarized in Supplementary Table 2 along with the rms roughnesses σ_{AFM}, and compared to the corresponding results of other measurement techniques.

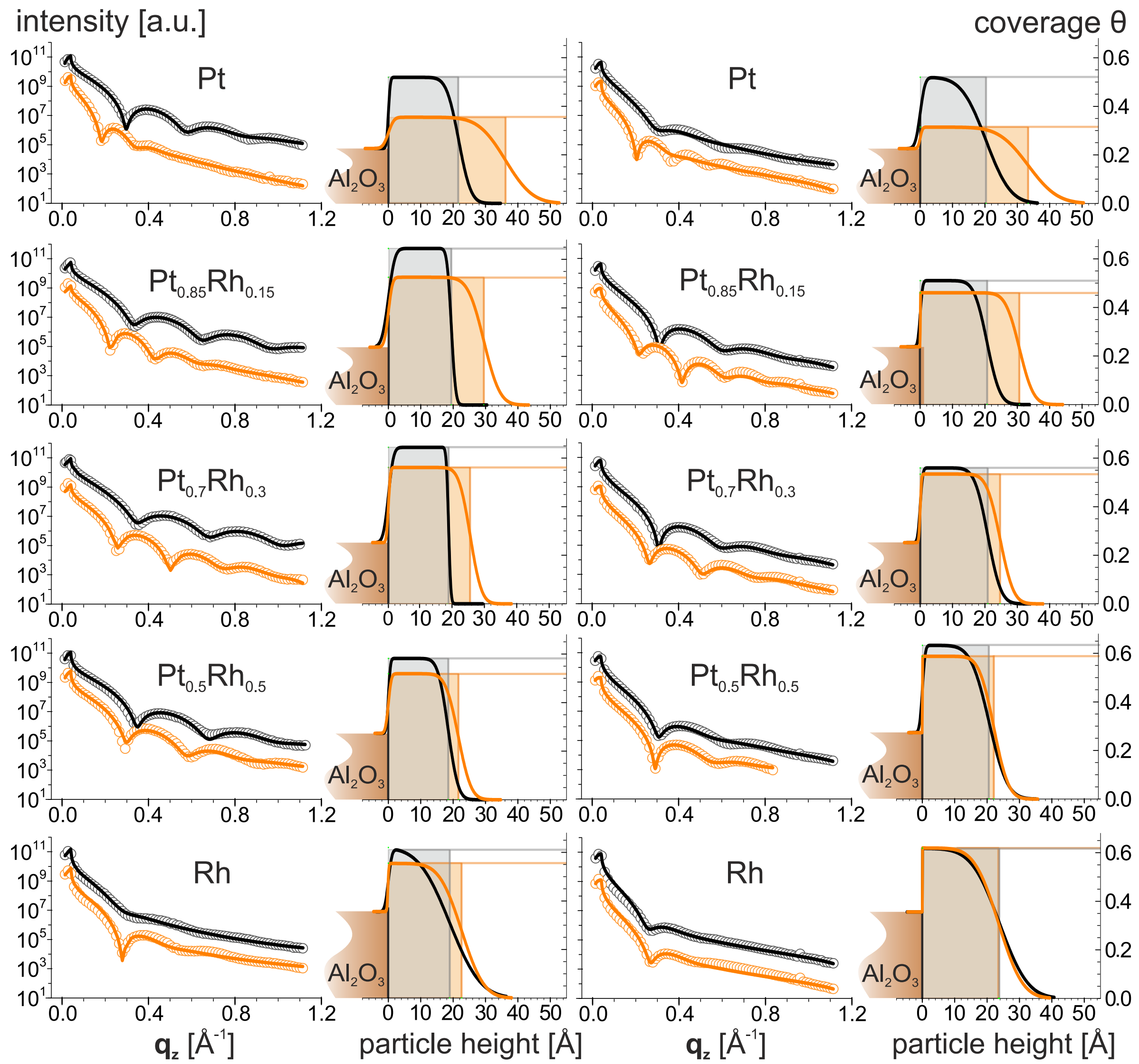

Supplementary Figure 6: **Juxtaposition of x-ray reflectivity results from two comparable samples:** X-ray reflectivity curves measured on the particles of varying Pt-Rh composition before (black) und during (orange) CO oxidation. Left column summarizes the results of the sample discussed in the main paper, right column the results of the second comparable sample discussed in the Supplementary Notes 6. Circles represent measured data, solid lines the fit to the data. To the right of the corresponding reflectivity curves are the electron density profiles obtained from the fits, which yield the surface fraction covered by nanoparticles.

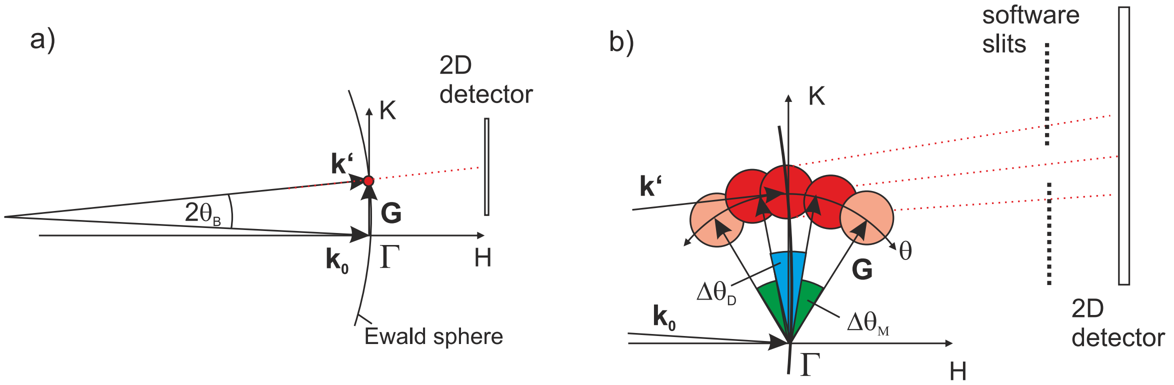

Supplementary Figure 7: **High energy x-ray diffraction geometry (a)**: Scattering geometry in the (H,K) surface plane **(b)**: zoom of the region close to the Bragg reflection, for details: see text.

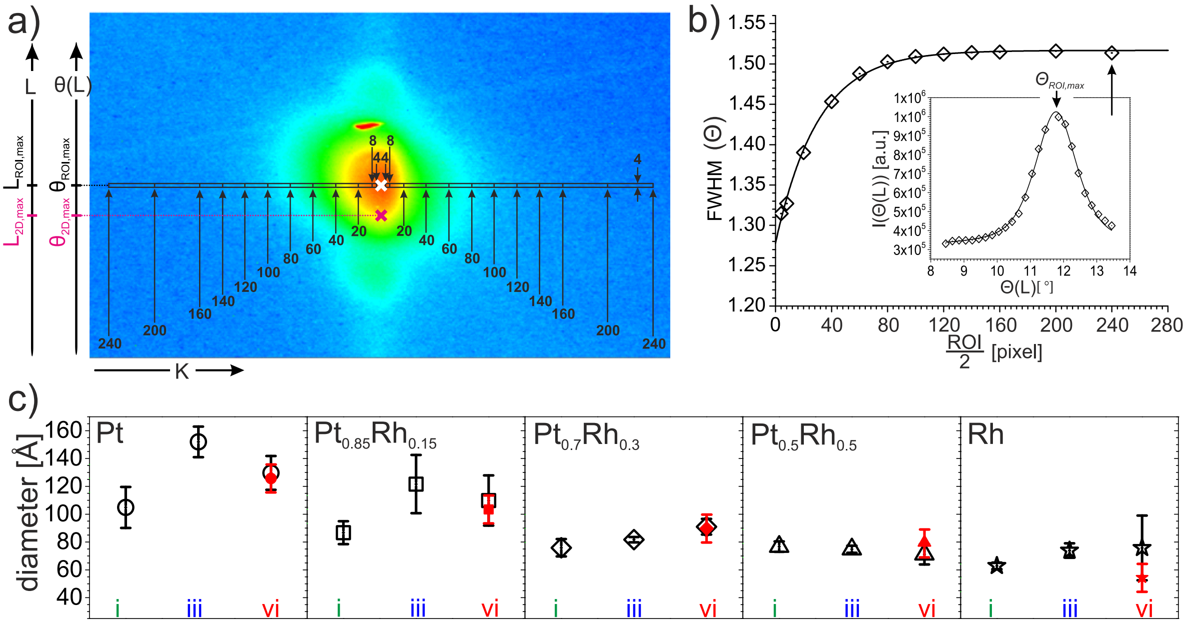

Supplementary Figure 8: **Particle diameter determination from θ-scans: (a)**: 2D image of the $Pt_{0.7}Rh_{0.3}$ particle Bragg peak, the peak maximum is denoted by the white cross. The black rectangles of varying horizontal size indicate regions of interest set during different θ-scans. The pairs of identical numbers, located symmetrically to the Bragg peak position, represent their respective horizontal borders. These numbers are proportional to the distance from the peak maximum position. The magenta cross correponds to the position of the θ-value $\theta_{2D,max}$ at which the 2D image was taken. **(b)**: Full Width at Half Maximums (FWHMs) obtained from θ-scans with different horizontal *ROI* sizes for the case shown in **a**. The inset displays the θ-scan performed with $\frac{ROI}{2}$=240 pixels, which was used for the intensity correction of the corresponding particle rod. **(c)**: Particle diameter for the different Pt-Rh compositions and reactivity conditions i, iii, vi as obtained from the θ-scans (black symbols). Red symbols indicate the particle diameters obtained from the AFM image analysis (see Supplementary Fig. 5) assuming an error of $\pm$10 Å.

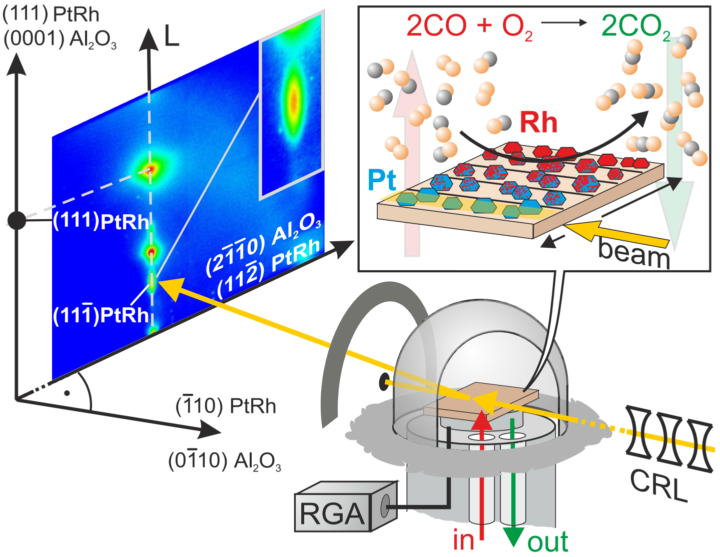

Supplementary Figure 9: **Measurement principle and probed reciprocal space plane for a second comparable sample:** the growth of the particle stripes along the substrate $(0\bar{1}10)$-direction allows to map the particle $(11\bar{1})$ Bragg peak, a close up of which is shown in the inset of the diffraction pattern.

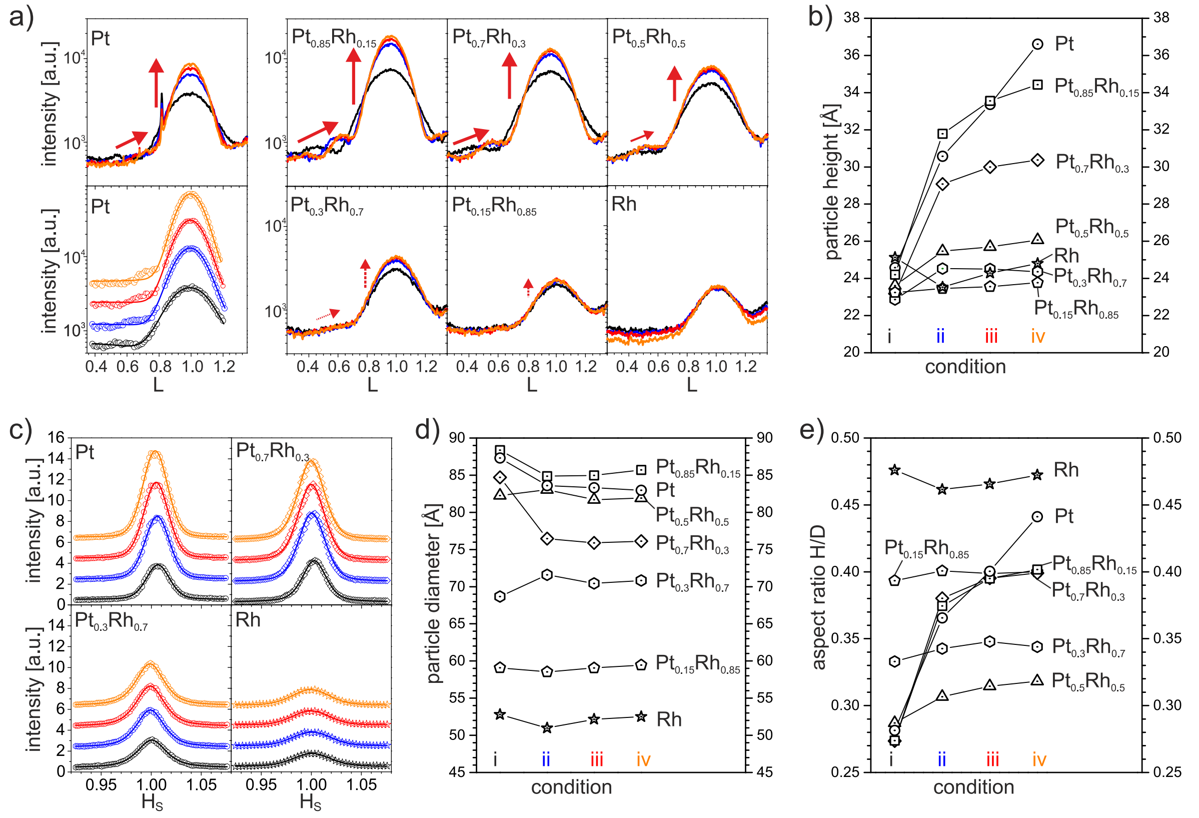

Supplementary Figure 10: **Particle heights and diameters and aspect ratios $\frac{H}{D}$ of a second comparable sample:** (**a**) linescans through the particle $(1\bar{1}1)$ Bragg peak extracted from the reciprocal space maps in L-direction for various Pt-Rh compositions and conditions (i (black): no O_2, 2 ml min^{-1} CO; ii (blue): 2 ml min^{-1} O_2, 10 ml min^{-1} CO, iii (red): 5 ml min^{-1} O_2, 10 ml min^{-1} CO, iv (orange): 8 ml min^{-1} O_2, 10 ml min^{-1} CO) at 550 K. The highlighted graphs show the linescans of the pure Pt particles as extracted from the 2D map (top) as well as drawn with an offset for the scans taken under the various conditions to show the Pseudo-Voigt fits (solid lines) to the data (circles). (**b**) particle heights determined from the L-scans considering the Full Width at Half Maximum of Pseudo-Voigt fits. (**c**) linescans (H_S-scans) extracted from the maps in horizontal direction (open circles: data; solid lines: Pseudo-Voigt fits) for the various concentrations and conditions drawn with an offset. In **c** the coordinate system of the particle surface unit cell (S) is used, where the probed direction $(100)_S$ correponds to $(11\bar{2})_B$ in the bulk (B) coordinate system. (**d**) particle diameters obtained from **c**. (**e**) aspect height to diameter ratios $\frac{H}{D}$ deduced from the data displayed in **b** and **d**.

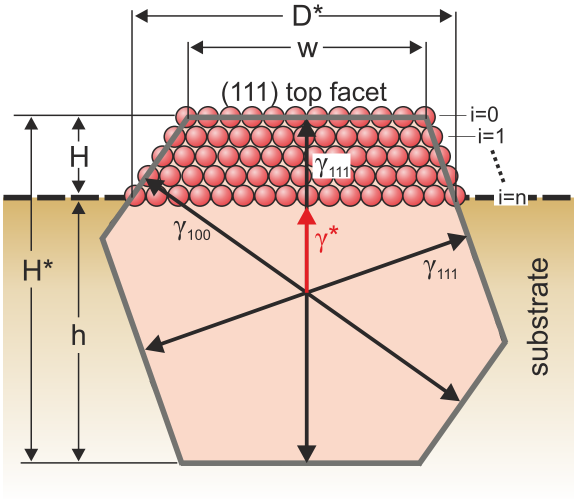

Supplementary Figure 11: **Wulff construction of a strongly truncated particle:** side view of a supported and strongly truncated (111)-oriented particle consisting of (111)- and (100)-type facets drawn according to the Wulff construction. H denotes the particle height, γ_{100} and γ_{111} the facet surface energies and γ^* the effective surface energy $\gamma^* = \gamma_{interface} - \gamma_{substrate}$ [1].

Supplementary Tables

stripe	cond.	b_0	b_1	t_{-2}	t_{-1}	t_0	t_1	t_2
**Pt**	**i**	-0.1076	-0.0240	-	-	-	-	-0.0644
	**iii**	-0.2488	-	-	-	-	-0.0116	-0.0583
	**vi**	-0.0971	-		-	-	0.0315	
**$Pt_{0.85}Rh_{0.15}$**	**i**	-0.1163	-	-	-	-	-	-0.0096
	**iii**	-0.1402	-0.0260	-	-	-	-	-0.1839
	**vi**	-0.1174	-0.0416	-	-	-	-	-0.0314
**$Pt_{0.7}Rh_{0.3}$**	**i**	-0.2074	-	-	-	-	-	-0.2568
	**iii**	-0.1375	-0.0061	-	-	0.0211	0.0374	0.1824
	**vi**	-0.1262	-	-	-	-	-	0.0000
**$Pt_{0.5}Rh_{0.5}$**	**i**	-0.1354	-	-	-	-	0.1184	0.1442
	**iii**	-0.1500	-0.0081	-	-	-	-	-0.0188
	**vi**	-0.1849	-	-	-	-	0.0055	0.0614
**Rh**	**i**	-0.1285	-			0.0870		
	**iii**	-0.1647	-			0.0889		
	**vi**	-0.1218	-			0.1540		

Supplementary Table 1: **Fitted parameters for the particle rod analysis:** the table contains results that were obtained based on particle model shapes with $g=\frac{\gamma_{(100)}}{\gamma_{(111)}}$=1.1. The colours of the table elements hold information whether for a certain Pt-Rh composition and condition (i, iii, vi) the respective occupancy of atomic layers b_0, b_1, t_{-2}, t_{-1}, t_0, t_1 and t_2 was allowed to vary in the fit (white elements) or whether it was kept fixed to its theoretical model value in order to maintain the physical constraints of the layer sequence (grey elements). The fitted occupancy values are displayed as black diamonds in Supplementary Fig. 3. The numbers of the table elements constitute the fitted displacement parameters of the corresponding layers, which represent the deviation (in Å) from the respective theoretical layer positions. A dash indicates that the displacement parameter was kept fixed during the fitting in order to fulfill physical constraints.

composition	H_{BP} [$\AA$]	H_{XRR} [$\AA$]	H_{AFM} [$\AA$]	σ_{XRR} [$\AA$]	σ_{AFM} [$\AA$]
**Pt**	41.64	36.179	16.872	6.583	6.615
$\mathbf{Pt}_{0.85}\mathbf{Rh}_{0.15}$	30.63	29.485	17.735	3.899	5.317
$\mathbf{Pt}_{0.7}\mathbf{Rh}_{0.3}$	27.26	25.247	9.287	2.764	3.098
$\mathbf{Pt}_{0.5}\mathbf{Rh}_{0.5}$	23.92	21.928	7.954	3.314	3.12
**Rh**	25.33	22.632	6.116	5.768	2.389

Supplementary Table 2: **Particle heights and roughnesses as obtained from different techniques:** the subscripts indicate from which data set the particle heights H and the rms roughnesses σ were retrieved ("BP": from particle Bragg peaks, see Fig. 3f of the main article; "XRR": data obtained from x-ray reflectivity fits, see Supplementary Fig. 6; "AFM": data obtained from the AFM measurements of Supplementary Fig. 5).

**composition**	**H** [$\AA$]	γ_{111} [eV Å^{-2}]	**g**=$\frac{\gamma_{100}}{\gamma_{111}}$	**w** [$\AA$], **h** [$\AA$]	$\mathbf{W}_{adh}$ [J m^{-2}]
**Pt**	38.61	0.093	0.9	w=84.31	2.23
			**1.1**	w=77.08	**1.98**
			1.3	w=81.90	1.86
$\mathbf{Pt_{0.85}Rh_{0.15}}$	29.39	0.09765	0.9	w=69.53	2.40
			**1.1**	w=64.73	**2.17**
			1.3	w=69.53	2.07
$\mathbf{Pt_{0.7}Rh_{0.3}}$	24.75	0.1023	0.9	w=59.65	2.53
			**1.1**	w=52.50	**2.24**
			1.3	w=59.65	2.20
$\mathbf{Pt_{0.5}Rh_{0.5}}$	20.12	0.1085	0.9	w=59.27	2.83
			**1.1**	w=52.16	**2.57**
			1.3	w=54.53	2.46
**Rh**	24.20	0.124	0.9	h=24.20	1.99
			**1.1**	h=22.00	**1.89**
			1.3	h=24.20	1.99

Supplementary Table 3: **Adhesion energies:** adhesion energies determined after sintering (condition vi) for the particles with different Pt-Rh compositions. In the case of Pt-containing particles equ. (1) and (2) were used, for the case of the pure Rh particles equ. (3) (for equations see Methods part of the main article). The values for the surface energies γ_{111} of the pure Pt and Rh particles were taken from [?], the surface energies $\gamma_{Pt_xRh_{1-x}}$ of the alloy particles Pt_xRh_{1-x} were calculated using the formula $\gamma_{Pt_xRh_{1-x}}$=$x\cdot$ $\gamma_{111,Pt}$ + (1-x) $\cdot$ $\gamma_{111,Rh}$.

Supplementary Notes

Supplementary Notes 1. High energy x-ray diffraction from nanoparticles

In conventional surface x-ray diffraction the data points along a CTR are measured with a point or 2D detector, while the rocking angle $\theta(L)$ of the sample needs to be varied according to each L-value along the rod. In our approach the nanoparticle rods were extracted from the reciprocal space maps shown in Fig. 2a of the main article which were measured at a fixed rocking angle $\theta_{2D,max}$ with a stationary detector. This implies that if all the nanoparticles on the sample were perfectly arranged - i.e. aligned along one in-plane direction determined by the substrate - they would give rise to diffraction at only one distinct point on the particle rods along the (111) direction, where its L-value would be determined by the corresponding $\theta(L)$.

However, on a real epitaxial nanoparticle system the particles display a certain angular distribution of 1°-2° around the surface normal (particle mosaicity) which compensates for the missing θ-rotation. This along with a flat Ewald sphere due to the usage of high photon energies results in the possibility of probing a vast area of reciprocal space with a single 2D snapshot at a fixed sample rocking angle θ_{meas}. This is illustrated in Supplementary Fig. 1, in which the orange particles are perfectly aligned with the support, whereas the blue and green particles display a slight rotation of $\pm\Delta\theta$ around the surface normal. The particles' angular distribution around their preferential orientation is well described by a Gaussian function, as is shown in the inset.

In x-ray diffraction, the orange particles just fulfill the diffraction condition for the particle Bragg peak, whereas the particles which are horizontally rotated give at the same time rise to diffraction above (blue) and below (green) this peak. Since the diffracted intensities are proportional to the number of scattering particles the particle rod intensity is also modulated by a Gaussian function, for which the linescans extracted from the maps need to be corrected.

In addition, the finite particle width D adds up to the enlargement of the probed area in reciprocal space. While an infinite particle diameter would result in sharp intersection points of the rods with the Ewald sphere (vertical dashed lines) the finite particle width makes them expand to $\Delta q_{xy}=\frac{2\pi}{D}$ as is indicated by the red, blue and green vertical bars. Furthermore, the non-zero energy distribution of the beam ΔE_0, leads to a broadening $\Delta k_0=\frac{\Delta E_0}{E_0}\cdot\mathbf{k_0}$ of the Ewald sphere which further enlarges the measurable range along the particle rod for a fixed rocking angle θ_{meas}. In Supplementary Fig. 1 its borders in L-direction are represented by red bars in the diffraction map; for the case of pure Pt particles this total range amounts to ΔL=3.89-1.45=2.44 r.l.u..

Supplementary Notes 2. Intensity correction of particle rods extracted from 2D maps

The particle rods which were used for the particle structure analysis were obtained from the 2D maps by extracting scans in L-direction through the corresponding main maximum of the particle Bragg peak. The scans were intensity-corrected for the background, the stationary sample and detector geometry and the Lorentz factor [2].

For **background correction** the positions above and below the Bragg peaks at which the particle signal subsides were determined. They served as intersection points for the linear background, which was subtracted from the extracted raw data.

In order to correct for the **stationary sample and detector** we had to compensate for the intensity modulation along the particle rod based on the angular distribution of the particles. Therefore θ-scans (rocking scans) were performed with a broad region of interest ("ROI", here: $\frac{ROI}{2}$=240 pixels, see Supplementary Fig. 8), i.e. detector area that was extended in horizontal direction. This ensured that the influence of the particle diameter D was fully included in the intensity distribution of the scan (see Supplementary Notes 4 for further explanation). Such a θ-scan is shown in the inset of Supplementary Fig. 8b, yielding the intensity distribution $I(\theta(L))$. The position of its maximum $\theta_{ROI,max}$ corresponds to the position on the particle rod given by the ROI position represented by the black box shown in Supplementary Fig. 8a. Its centre coincides with the Bragg peak maximum indicated by the white cross.

Since the experimental 2D images, from which the L-scans were extracted, were taken at a slightly different θ-value, which we call $\theta_{2D,max}$ in the following, we had to compensate for this offset by expressing $\theta(L)$ as $\theta^*(L)$=$\theta(L)$-$\Delta\theta$, where $\Delta\theta$=$\theta_{2D,max}$-$\theta_{ROI,max}$ (see Supplementary Fig. 8a). Thus, the correction factor F_θ for the angular distribution of the particles reads:

$$F_\theta = \frac{I(\theta_{2D,max})}{I(\theta^*(L))}, \tag{1}$$

where F_θ=1 if $\theta^*(L)$=$\theta_{2D,max}$, otherwise F_θ<1. Taking moreover the **Lorentz factor** F_L=$\frac{1}{sin(\gamma)}$, which corrects for the stationary geometry of sample and detector [2], into account, the total correction factor F amounts to:

$$F = F_\theta \cdot F_L = \frac{I(\theta_{2D,max})}{I(\theta^*(L)) \cdot sin(\gamma)}. \tag{2}$$

This expression was multiplied by the background-subtracted L-scans in order to obtain the intensity-corrected scans that were used for the structure analysis of the nanoparticles.

Supplementary Notes 3. Particle rod fit results

In order to determine which of the particle shape models shown at the top of Supplementary Figs. 2 and 3 fits the data best, they were all used as underlying fit models and compared with each other. Supplementary Fig. 2 yields an overview of the figures of merit χ^2 obtained for the different models for all Pt-Rh compositions and for the sample conditions i, iii and vi. The number of used fit parameters for a certain condition and Pt-Rh composition was the same for all tested models in order to ensure a comparability, but could vary from one condition or particle stripe to the next. It was chosen such that the necessary number of fit parameters was used while ensuring that none of the fits ran into unphysical values (see Methods part in the main article).

The comparison shows that independent of composition and condition the particle shapes with a lower g-value are favoured. This is in agreement with DFT-calculations that suggest for all types of conditions particle shapes with a g-value ranging between 0.9 and 1.3 [3]. We find that for conditions with increased catalytic activity (especially for condition vi) this trend towards smaller g-values and thus larger (100)-type facets becomes all the more pronounced. A tentative explanation could be that with higher oxygen partial pressures and thus with increasing catalytic activity more oxides form on the particle facets, which is easier on the more open (100)-type facets. In most cases the particle models with a surface energy ratio between g=0.9 and g=1.3 and the model of a truncated sphere yielded the lowest χ^2 values. This indicates that the particles might have a rounder particle shape and/or higher indexed facets not considered in the initial particle shape models. Particle shapes with a high g-values like 1.5 or $\sqrt{3}$ could along with the closed layer model be ruled out. Thus, although the juxtaposition does not allow to determine a specific particle shape, certain trends can be deduced.

For a refined fitting the underlying particle model shape with a surface energy aspect ratio of g=1.1 was used. The fits to the data are shown as solid lines in Fig. 3a-e of the main article. The fitted occupancy values for the different compositions and conditions are represented by black diamonds in Supplementary Fig. 3, the fit values for the displacement parameters are summarized in Supplementary Table 2. From the results shown in Supplementary Fig. 3 it can be deduced that a certain particle height distribution is present, which is mirrored by the smearing out of the occupancy values. This trend is in accordance with the smearing out of the electron density profile obtained from x-ray reflectivity data displayed in Supplementary Fig. 6 and is found to be stronger for the sintered particles. The occupancy values at the interface are strongly reduced which implies that not all atoms at the interface are located at their correct crystal sites. This can either be explained by strain effects or by the assumption of particles sitting on substrate defects like steps and edges.

As main conclusion one can say that within the error bars the same fit result for the occupancy values is obtained for all surface energy ratios $g=\frac{\gamma_{100}}{\gamma_{111}}$ ranging between 0.9 and 1.3. Even when using the shape of a truncated sphere as underlying particle model the same occupancy values are received from the fit,

implying that the particle shape is not spherical but rather decorated by higher indexed facets.

Supplementary Notes 4. Determination of particle diameters from rocking scans

In this work rocking scans (here: θ-scans) were not only used to obtain the angular distribution of the particles for correcting the intensity modulation along the particle rods, as explained in Supplementary Notes 2, but also to gain information on the particle diameters under different conditions of catalytic activity. How an upper limit of the average particle diameter can be deduced from θ-scans will be addressed in the following.

In Supplementary Fig. 7a the high energy x-ray diffraction geometry is plotted for the in-plane component of the $(3\bar{1}1)$ reflection (which is the $(2\bar{2}0)$ reflection) in the (H,K) plane parallel to the sample surface. $\mathbf{k_0}$ and $\mathbf{k'}$ denote the incident and the scattered wave vectors with $\mathbf{k_0} = \mathbf{k'} = \frac{2\pi}{\lambda}$ (with x-ray wave length λ=0.1575 Å). The in-plane Bragg angle θ_B amounts in our case to 3.26°. It is related via $Q = \frac{4\pi}{\lambda}\sin\theta_B$ to the magnitude of the scattering vector $\mathbf{Q}$=$\mathbf{k'}$-$\mathbf{k_0}$. The reciprocal lattice vector $\mathbf{G}$ points from the origin of reciprocal space Γ to the reciprocal lattice point $(3\bar{1}1)$. Due to the (average) finite diameter $\overline{D}$ of the nanoparticles, the Bragg reflection (circle in Supplementary Fig. 7) is broadened in the H and K direction by $\Delta H = \Delta K = \frac{2\pi}{\overline{D}}$ (thereby assuming a round particle shape and for the moment negligible inhomogeneous strain broadening of the Bragg reflections).

Diffraction occurs, when the reciprocal lattice is intersecting the Ewald sphere, and the intersection point defines the direction of the diffracted wave vector $\mathbf{k'}$. This situation is illustrated in Supplementary Fig. 7b, representing a close-up of the Bragg peak region: due to the high energies and the correspondingly small Bragg angles, in a rocking (θ) scan with rotation axis perpendicular to the (H,K) plane, the width of the Bragg peak is probed along H and the corresponding peak width is given by $\Delta\theta_{\overline{D}} = \frac{d_{2\bar{2}0}}{\overline{D}}$, where $d_{2\bar{2}0}$ denotes the lattice spacing of the $(2\bar{2}0)$ planes. For a real nanoparticle system, a certain angular distribution of the particles (mosaicity) with in-plane mosaic spread $\Delta\theta_M$ will be present, which is superimposed to $\Delta\theta_{\overline{D}}$. In a rocking scan with open detector slits (i.e. integration of the Bragg peak over K on the 2D detector), the peak width $\Delta\theta_{M,\overline{D}}$ is thus the convolution of mosaicity and particle broadening effect: $\Delta\theta_{M,\overline{D}}$=$\sqrt{(\Delta\theta_M)^2 + (\Delta\theta_{\overline{D}})^2}$. Please note that in our case, $\overline{D}$ can not be directly obtained from ΔK on the 2D diffraction images because the $(3\bar{1}1)$ reflection exhibits in the K-direction a significant broadening due to a lattice parameter distribution in the nanoparticle ensemble. The broadening along H (for K=0) is however insensitive to such inhomogeneous strain effects.

To obtain the pure mosaicity contribution ($\Delta\theta_M$), successive θ-scans were performed with differently set regions of interest ("*ROIs*", software slits in Supplementary Fig. 7b), which were centered around the Bragg peak maximum and over which the intensity was integrated during the scans. This is illustrated

in Supplementary Fig. 8a in which the sizes of the different ROIs are indicated by arrows which are drawn with respect to the Bragg peak maximum indicated by the white cross. The numbers correspond to the ROI width in pixels, where all ROIs share the same vertical height of 4 pixels.

Supplementary Fig. 8b shows the FWHMs of the performed θ-scans as obtained as a function of the horizontal ROI sizes. The FWHMs were obtained by fitting the respective θ-scans with Pseudo-Voigt functions. An example is shown in the inset of Supplementary Fig. 8b which represents the fitted θ-scan obtained for the $Pt_{0.7}Rh_{0.3}$ particles and the largest horizontal ROI size of ROI=2·240 pixels.

When the ROI size along K is decreased, the contribution of smaller particles within the size distribution to the Bragg peak signal is decreased accordingly (because smaller particles give rise to wider Bragg reflections), and in the θ-scan an effectively larger average particle size $\overline{D}$ is probed. In the limit of zero ROI width, an upper limit for θ_M can thus be determined (and a lower limit for $\delta\theta_{\overline{D}}$). An upper limit for the average particle diameters $\overline{D}$ was finally obtained via $\overline{D} = \frac{d_{(2\bar{2}0)}}{\Delta\theta_{\overline{D}}}$, where $\Delta\theta_{\overline{D}}=\sqrt{(\Delta\theta_{M,\overline{D}})^2 - (\Delta\theta_M)^2}$.

For the case of the example in Supplementary Fig. 8 we obtain 97 Å, in good agreement with the AFM value of 90 Å (see Supplementary Figs. 8c and 5b). The results for the upper limits of the average diameters $\overline{D}$ are summarized in Supplementary Fig. 8c for the various Pt-Rh compositions and conditions, where systematic error bars obtained from the fits are included. They show that the particle diameters neither vary to a great extent nor in any systematic way under the different conditions of catalytic activity. This finding is in line with the results gained for a second comparable sample (see Supplementary Notes 6) for which the particle diameters were also found to stay rather constant independent of the sample condition. Furthermore, we find a Pt-Rh-composition-dependent gradient in the particle diameter with pure Pt particles displaying the largest, pure Rh particles the smallest diameter.

Supplementary Notes 5. Determination of particle diameters from AFM measurements

Supplementary Fig. 5a shows AFM data of the different particle stripes performed under UHV conditions after the CO oxidation experiment at 550 K discussed in the main article, and after additional CO oxidation reactions at 670 K, which according to our x-ray data did not lead to further pronounced particle aspect ratio changes.

Supplementary Fig. 5b displays the particle diameter distribution as obtained from the microscopy data fitted with Gaussians. The maxima of the fits correspond to the respective particle diameters D^*_{AFM} from which the real particle diameter D_{AFM} was obtained via D_{AFM}=D^*_{AFM}-20 Å, taking the tip radius into account. The values of the diameters D_{AFM} are represented by the red symbols in Supplementary Fig. 8c. They are in good agreement with the diameters obtained from the rocking

scans. Supplementary Fig. 5b illustrates moreover a composition-dependence of the particle densitiy: while there are only few - but large - pure Pt particles, the particle number rises with increasing Rh composition at the expense of the particle diameter.

Supplementary Fig. 5c reveals the height histogrammes as obtained from the AFM measurements displayed in Supplementary Fig. 5a. The deduced average heights along with the rms roughnesses are summarized in Supplementary Table 2. They reveal that the average height of the particles after CO oxidation decreases with increasing Rh content, which implies that the sintering in height scales with the Pt-content of the particles. The results are hence in line with the ones obtained from the particle Bragg peak analysis and the x-ray reflectivity results, also to be found in Supplementary Table 2. The discrepancy between the results obtained from AFM and the x-ray-based techniques can be traced back to the large tip radius and the relatively high particle coverage on the sample surface, which prevents the tip from reaching the bare substrate level, which leads to a generic underestimation of the particle height in the AFM data. Both, AFM and x-ray reflectivity results show a relatively high rms roughness for the strongly sintered Pt-rich particles.

Supplementary Notes 6. Data reproducibility (results of a second comparable sample)

The reproducibility of the main article's data was confirmed by results obtained from a second comparable sample. Also in this case the combinatorial sample consisted of $Al_2O_3(0001)$-supported alloy particles of the same Pt-Rh compositions as discussed in the main article (Pt, $Pt_{0.85}Rh_{0.15}$, $Pt_{0.7}Rh_{0.3}$, $Pt_{0.5}Rh_{0.5}$, Rh), but included in addition two stripes with higher Rh content ($Pt_{0.3}Rh_{0.7}$, $Pt_{0.15}Rh_{0.85}$). Contrary to the sample discussed in the main article the particle stripes were grown along the $(0\bar{1}10)$-direction of the substrate. This allowed for monitoring reciprocal space maps including the particle $(11\bar{1})$ Bragg peak, as can be inferred from Supplementary Fig. 9.

The sample was studied under the same conditions (p_{tot}=200 mbar, T=550 K) employing the same experimental set-up (combined high energy grazing incidence x-ray diffraction at 78.8 keV and in-situ mass spectrometry). Also in this case the stepwise increase of O_2 pressure (investigated steps: i: no O_2, 2 ml min^{-1} CO; ii: 2 ml min^{-1} O_2, 10 ml min^{-1} CO; iii: 5 ml min^{-1} O_2, 10 ml min^{-1} CO; iv: 8 ml min^{-1} O_2, 10 ml min^{-1} CO) was accompanied by an increase in the CO_2 production, where for each step the reciprocal space maps were monitored for the different Pt-Rh compositions.

Linescans running in vertical (see Supplementary Fig. 10a) and horizontal (see Supplementary Fig. 10c) directions through the particle $(11\bar{1})$ Bragg peaks were extracted from the maps and fitted using Pseudo-Voigt functions in order to obtain the corresponding particle heights and diameters under the various conditions from the corresponding full widths at half maxima (FWHMs). Accordingly, the di-

ameters were determined via $D = \frac{a^*}{\Delta q_{\|}}$, where a*=$\frac{2\pi \cdot 2\sqrt{2}}{\sqrt{3} \cdot a_{fcc}}$ and $\Delta q_{\|}$ corresponds to the FWHM of the in-plane scan through the particle Bragg peak. The FWHM-analysis, yielding the domain size and thus the lower limit for the corresponding particle diameter, is feasible for this sample as the q-value of the particle $(11\bar{1})$ peak is lower compared to the one of the $(3\bar{1}1)$ peak probed for the sample discussed in the main article. Thus strain broadening effects due to particles growing commensurately on the alumina-substrate become negligible and the lower limit obtained from the FWHM-analysis close to the correct value for the average particle diameter. The main results on the composition- and condition-dependent particle heights and diameters are summarized in Supplementary Fig. 10a-e.

As can be inferred from Supplementary Fig. 10b the particles of the various Pt-Rh compositions had the same initial height of about 24 Å. In accordance with the data set discussed in the main article the height increase in the transition to higher catalytic activity conditions was most pronounced for pure Pt particles and was progressively reduced for particles with increasing Rh content. Also for this second sample the diameter was found to be composition-dependent with a tendency towards smaller diameters with increasing Rh composition (see Supplementary Fig. 10d). As the total annealing time of the Pt-rich particles during sample preparation was shorter for this sample compared to the sample discussed in the main article (1 hours vs. 5 hours), the Pt-rich particles on this second sample display a smaller diameter as they may have undergone less sintering during particle growth. Also here it was found that all particle diameters stayed constant in the course of the experiment. The corresponding height to diameter aspect ratios can be found in Supplementary Fig. 10e. In agreement with the result of the first sample the aspect ratio of the Pt-rich particles increased towards the value of the pure Rh particles, which here also amounted to about 0.45. These findings underline the assumption that the flatter Pt-rich particles undergo activity-induced shape changes towards a more three-dimensional equilibrium shape.

Supplementary Fig. 6 shows a juxtaposition of x-ray reflectivity results obtained for both samples measured before (black) and during high catalytic activity (orange). From the fit results it can be conluded that for both samples the particle height of the Pt-rich particles increased at the expense of the particle coverage on the surface, which was found to be more pronounced the higher the Pt content of the particles.

Supplementary References

1. Hansen, K. H. et al. Palladium nanocrystals on Al_2O_3: structure and adhesion energy. *Phys. Rev. Lett.* **83**, 4120-4123 (1999).

2. Vlieg, E. Integrated intensities using a six-cirlce surface x-ray diffractometer. *J. Appl. Cryst.* **30**, 532-543 (1997).

3. Seriani, N., Mittendorfer, F. Platinum-group and noble metals under oxidizing conditions. *J. Phys.: Condens. Matter* **20**, 184023-184033 (2008).
